# Supplementary material for: Synthetic gene circuits that selectively target RAS-driven cancers
Source: eLife. 2026 Feb 24;14:RP104320. doi: 10.7554/eLife.104320 (PMC12931925; doi:10.7554/eLife.104320)
Supplement: Figure 2—source data 1. [file elife-104320-fig2-data1.docx]

| **Name (& Database Identifier)** | **Sequence** | **Position**  **(0- based)** | **Strand** | **Score** | **p-value** | **E- value** |
| --- | --- | --- | --- | --- | --- | --- |
| Creb1 (PAZAR, 81646) | GTGTTCTGGCGGCAA | 213 | - | 15.05 | 0.0009 | 1.05 |
| Creb1 (PAZAR 81646) | GTGTTCTGGCGGCA | 214 | - | 15.96 | 0.000425 | 0.50 |
| c-Myc:Max (M00615) | CAAGAATCACGTACTGCAGC | 314 | - | 5.83 | 0.001 | 1.16 |
| c-Myc:Max (M00118) | TCGCCTCGTGCTTG | 396 | + | 3.6 | 0.000675 | 0.79 |
| Myc (MA0147.1) | AGCACGAGGC | 398 | - | 9.23 | 0.00045 | 0.53 |
| MYC::MAX (MA0059.1) | AAGCACGAGGC | 398 | - | 7.98 | 0.0006 | 0.70 |
| c-Myc:Max (M00123) | AAGCACGAGGC | 398 | - | 12.54 | 0.000975 | 1.14 |
| Mycn (MA0104.1) | CTCGTG | 400 | + | 7.14 | 0.000525 | 0.62 |
| Myc (MA0147.1) | CGCACGCGGC | 439 | - | 10.18 | 0.0003 | 0.35 |
| c-Myc:Max (M00123) | GCGCACATGTT | 632 | + | 12.35 | 0.001 | 1.17 |
| Myc (MA0147.1) | CGCACATGTT | 633 | + | 9.8 | 0.000325 | 0.38 |
| Mycn (MA0104.1) | CACATG | 635 | + | 8.11 | 0.000525 | 0.62 |
| SRF (PAZAR 6722) | CCGAACATG | 637 | - | 13.33 | 0.00015 | 0.176 |
| Srf (PAZAR 20807) | CATGTTCGGC | 637 | + | 12.61 | 0.000875 | 1.03 |
| Tax/CREB (M00115) | GTGACTCACCCGCCC | 862 | - | 7.91 | 0.000525 | 0.61 |
| CREB (M00113) | TGGGTGACTCAC | 868 | - | 9.39 | 0.000625 | 0.73 |
| Jun/Fos Heterodimer  (UP00425.Jun+Fos Heterodimer.primary) | CGGGTGAGTCACCC | 865 | + | 13.39 | 0.000025 | 0.0292 |
| Fos (MA0099.1) | GTGAGTCA | 868 | + | 12.61 | 0.000025 | 0.0294 |
| AP1 (Jun family) CRE (ORegAnno 9606 103). | TGACTCAC | 868 | - | 15.76 | 0.00055 | 0.65 |
| Fos (MA0099.1) | GTGACTCA | 869 | - | 12.72 | 0.000025 | 0.0294 |
| JUN (PAZAR 3725) | TGACTCA | 869 | - | 14.16 | 0.000075 | 0.088 |
| Mycn (MA0104.1) | CACATG | 919 | - | 8.11 | 0.000525 | 0.62 |
| JUN (PAZAR, 3725) | GTGACTCC | 922 | + | 13.72 | 0.0002 | 0.235 |
| JUN (PAZAR, 3725) | TGACTCC | 923 | + | 14.29 | 0.000075 | 0.088 |
| AP1 (Jun family) CRE (ORegAnno 9606 103) | TGACTCC | 923 | + | 15.89 | 0.00055 | 0.65 |
| Srf (UP00077.Srf.primary) | CACCTGAAATGGAA | 1162 | - | 8.26 | 0.0003 | 0.35 |
| ELK4 / Sap1 (MA0076.1) | ACCTGAAAT | 1166 |  | 7.16 | 0.000925 | 1.09 |

**Figure 2 – Source Data 1 | Prediction of MAPK transcription factor binding sites in EF1a – numerical values.** List of all predicted binding sites of Myc, AP1 (Fos/Jun), SRF, CREB, and Ets/Elk transcription factors with LASAGNA 2.0 using the TRASFAC TFBS search, which are visualized in Figure 2 – figure supplement 1. For each binding site the sequence, position in EF1a, strand, binding strength (score), statistical significance (p-value), and expected number of random hits with similar scores in the data set (e-value) are provided.
