# Supplementary material for: Synthetic gene circuits that selectively target RAS-driven cancers
Source: eLife. 2026 Feb 24;14:RP104320. doi: 10.7554/eLife.104320 (PMC12931925; doi:10.7554/eLife.104320)
Supplement: Figure 5—source data 1. [file elife-104320-fig5-data1.docx]

**Figure 5 – Source Data 1:** Numerical values of flow cytometry histograms shown in Figure 5d

|  | **Results in HEK293 with KRAS^G12D^**  (Results from concatenated triplicates) | | | **Results in HEK293 with KRAS^WT^**  (Results from concatenated triplicates) | | |
| --- | --- | --- | --- | --- | --- | --- |
| **Circuit ID** | Mean mCerulean expression | Freq. of Parent [% of all measured cells] | Number of mCerulean positive cells | Mean mCerulean expression | Freq. of Parent  [% of all measured cells] | Number of mCerulean positive cells |
| RAS_Sensor_F.L.T. | 1361 | 1.9 | 666 | 1013 | 0.5 | 119 |
| PY2_NarL-F.L.T. | 1463 | 4.2 | 1456 | 93 | 0.2 | 14 |
| PY2_NarX_F.L.T. | 1763 | 4.0 | 1587 | 297 | 0.2 | 24 |
| PY2_all_F.L.T. | 1707 | 7.1 | 2547 | 191 | 0.3 | 26 |
