# Supplementary material for: Synthetic gene circuits that selectively target RAS-driven cancers
Source: eLife. 2026 Feb 24;14:RP104320. doi: 10.7554/eLife.104320 (PMC12931925; doi:10.7554/eLife.104320)
Supplement: Figure 5—source data 2. [file elife-104320-fig5-data2.docx]

**Figure 5 – Source Data 2:** Tested regression models for Figure 5c to select the independent variables and assess the interaction between response element and expressed parts.

|  | Model 1 | Model 2 | Model 3 (Selected Model) |
| --- | --- | --- | --- |
| Independent Variable(s) used to describe the response element (RE) | RE | RE_NarX  + RE_NarL | RE_NarX +  RE_NarL_TAD |
| Description/ Assumptions | The effect of the response elements is independent of what part is expressed. | The effect of response elements depends on whether they are used to express the NarX fusion proteins (RE_NarX) or NarL protein (RE_NarL). | The effect of response elements depends on whether they are used to express the NarX fusion proteins or NarL protein.  Additionally, the effect of REs depends on the TAD fused to NarL (RE_NarL_TAD). |
| Complete Formula used in regression model | Log(RASmut_RU) ~ RASmut_mCherry + **RE** + BD + TAD + Linker + Conc_NarX + Conc_NarL | Log(RASmut_RU) ~ RASmut_mCherry + **RE_NarX + RE_NarL** + BD + TAD + Linker + Conc_NarX + Conc_NarL | Log(RASmut_RU) ~ RASmut_mCherry + **RE_NarX + RE_NarL_TAD** + BD + Linker + Conc_NarX + Conc_NarL |
| ON-State Model (HEK^G12D^): | Pearson R^2^ = 0.44 Spearman = 0.62 | Pearson R^2^ = 0.45 Spearman = 0.63 | Pearson R^2^ = 0.68 Spearman = 0.82 |
| OFF-State Model (HEK^WT^): | Pearson R^2^ = 0.44 Spearman = 0.67 | Pearson R^2^ = 0.53 Spearman = 0.73 | Pearson R^2^ = 0.61 Spearman = 0.78 |
| Dynamic Range Model (HEK^G12D^/HEK^WT^): | Pearson R^2^ = 0.44 Spearman = 0.66 | Pearson R^2^ = 0.50 Spearman = 0.70 | Pearson R^2^ = 0.60 Spearman = 0.75 |
