# Supplementary material for: Synthetic gene circuits that selectively target RAS-driven cancers
Source: eLife. 2026 Feb 24;14:RP104320. doi: 10.7554/eLife.104320 (PMC12931925; doi:10.7554/eLife.104320)
Supplement: Figure 6—source data 1. [file elife-104320-fig6-data1.docx]

**Figure 6 – Source Data 1:** Numerical values of flow cytometry histograms shown in Figure 6e

|  | **Results in HCT-116 wildtype**  (Results from concatenated triplicates) | | | **Results in HCT-116 knock out**  (Results from concatenated triplicates) | | |
| --- | --- | --- | --- | --- | --- | --- |
| **Circuit ID** | Mean mCerulean expression | Freq. of Parent [% of all measured cells] | Number of mCerulean positive cells | Mean mCerulean expression | Freq. of Parent  [% of all measured cells] | Number of mCerulean positive cells |
| PY2_NarL-F.L.T._3xrL | 2553 | 21.9 | 2766 | 1067 | 0.9 | 157 |
| RAS_Sensor_F.L.T. | 2226 | 13.3 | 2427 | 469 | 1.0 | 185 |
| PY2_NarX_F.L.T. | 2270 | 6.6 | 1703 | 347 | 0.4 | 81 |
| PY2_all_F.L.T | 3337 | 9.1 | 1361 | 1362 | 0.5 | 421 |
| PY2_NarL-F.L.T. | 4725 | 10 | 1196 | 1178 | 0.2 | 317 |
