# Supplementary material for: Synthetic gene circuits that selectively target RAS-driven cancers
Source: eLife. 2026 Feb 24;14:RP104320. doi: 10.7554/eLife.104320 (PMC12931925; doi:10.7554/eLife.104320)
Supplement: Supplementary file 3. [file elife-104320-supp3.docx]

**Supplementary File 3:** Cell line and culturing details.

| **Cell Line** | **Medium** | **Spitting Ratio** | **Provider** | **Catalogue Number** | **RRID** | **RAS status^1^** |
| --- | --- | --- | --- | --- | --- | --- |
| **A-549** | RPMI-1640 | 1:2 - 1:15 | DSMZ | ACC 107 | CVCL_0023 | KRAS^G12S^ (homozygous) |
| **AsPC-1** | RPMI-1640 | 1:2 - 1:6 | ATCC | CRL-1682 | CVCL_0152 | KRAS^G12D^ (homozygous) |
| **HCT-116** | McCoy`s 5A | 1:2 - 1:20 | Abcam | ab288559 | CVCL_0291 | KRAS^G13D^ (heterozygous) |
| **HCT-116 KRAS knock out** | McCoy`s 5A | 1:2 - 1:15 | Abcam | ab276083 | CVCL_B1DS | KRAS knock out |
| **HEK293** | DMEM | 1:2 - 1:20 | Invitrogen | 11631-017 | CVCL_0045 | wildtype |
| **HeLa** | DMEM | 1:2 - 1:6 | ATCC | CCL-2 | CVCL_0030 | wildtype |
| **HT-29** | McCoy`s 5A | 1:2 - 1:8 | ATTC | HTB-38 | CVCL_0320 | Wildtype RAS; BRAF^V600E^ (heterozygous) |
| **Igrov-1** | DMEM | 1:2 - 1:6 | Sigma-Aldrich | SCC203 | CVCL_1304 | wildtype |
| **K-562** | RPMI-1640 | 10^5^ - 10^6^ cells per mL | ATCC | CCL-243 | CVCL_0004 | BCR-ABL |
| **LoVo** | F-12K | 1:2-1:10 | ATCC | CCL-229 | CVCL_0399 | KRAS^G13A^ (heterozygous) |
| **MCF-7** | DMEM | 1:2-1:6 | ATCC | HTB-22 | CVCL_0031 | wildtype |
| **SKOV-3** | McCoy`s 5A | 1:2-1:6 | ATCC | HTB-77 | CVCL_0532 | wildtype |
| **SW480** | DMEM | 1:2 - 1:8 | ATCC | CCL-228 | CVCL_0546 | KRAS^G12V^ (homozygous) |
| **SW620** | DMEM | 1:2 - 1:10 | ATCC | CCL-227 | CVCL_0547 | KRAS^G12V^ (homozygous) |

^1^Bairoch A. The Cellosaurus, a cell line knowledge resource. J. Biomol. Tech. 29:25-38 (2018).

DOI: 10.7171/jbt.18-2902-002; PMID: 29805321; PMCID: PMC5945021
