# Supplementary material for: Synthetic gene circuits that selectively target RAS-driven cancers
Source: eLife. 2026 Feb 24;14:RP104320. doi: 10.7554/eLife.104320 (PMC12931925; doi:10.7554/eLife.104320)
Supplement: Supplementary file 6. [file elife-104320-supp6.docx]

**Supplementary File 6 – Table A**: Seeding and transfection conditions in different cancer cell lines (Figure 7 & 8d-l)

| Cell line | Seeding (24-well)  [10^3^ cells/well] | Transfection Reagent | DNA:Reagent ratio  [µL/µg] | DNA amount |
| --- | --- | --- | --- | --- |
| A-549 | 77 | Viafect  (Promega, cat #E4981) | 8 | 1.5x |
| AsPC-1 | 89 | Fugene HD  (Promega, cat #E2311) | 3.5 | 1.5x |
| HCT-116 | 59 | Fugene HD | 3 | 0.5x |
| HeLa | 59 | Fugene HD | 3 | 1.5x |
| HT-29 | 71 | Lipofectamine 3000  (invitrogen, cat #L3000008) | 2 | 1.5x |
| Igrov-1 | 45 | Fugene HD | 3 | 1x |
| K-562 | 119 | Lipofectamine 2000  (invitrogen, cat #11668019) | 3 | 1.5x |
| LoVo | 89 | Fugene HD | 3 | 1.5x |
| MCF-7 | 59 | Fugene HD | 3 | 1.5x |
| SKOV-3 | 30 | Fugene HD | 3 | 1.5x |
| SW480 | 77 | Fugene HD | 3 | 1.5x |
| SW620 | 101 | Lipofectamine 3000 | 2 | 1.5x |

**Supplementary File 6 – Table B**: Seeding and transfection conditions for all experiments (Figure1-6e)

| Cell line | Seeding  96-well  [10^3^ cells/ well] | Seeding  24-well  [10^3^ cells/ well] | Seeding  6-well  [10^3^ cells/ well] | Transfection Reagent | DNA:Reagent ratio [µg/µL] | DNA amount |
| --- | --- | --- | --- | --- | --- | --- |
| HEK293 | 20 | 75 | 400 | Lipofectamine 2000 | 2 | 1x |
| HCT-116 wildtype | 10 | 60 | - | Fugene | 3 | 0.25-1x* |
| HCT-116 KRAS k.o. | 20 | 100 | - | Fugene | 3 | 1x |

*see also Supplementary File 4
