# Supplementary material for: Synthetic gene circuits that selectively target RAS-driven cancers
Source: eLife. 2026 Feb 24;14:RP104320. doi: 10.7554/eLife.104320 (PMC12931925; doi:10.7554/eLife.104320)
Supplement: Supplementary file 7. [file elife-104320-supp7.docx]

**Supplementary File 11**: Segmentation parameters for image analysis using Agilent eSight in Fig.8

| Cell Line –  Channel | Segmentation Adjustment (Background <-> Cell) | Cleanup (Hole Fill) | Area Filter  (Minimal Cell Size) | Edge Sensitivity |
| --- | --- | --- | --- | --- |
| HCT-116 –  Bright field | 0.6 | 0 | 1000 | - |
| HCT-116 –  Red Fluorescence | 0.6 | 500 | 100 | 0 |
| Igrov-1 –  Bright field | 0.9 | 0 | 1500 | - |
| Igrov-1 –  Red Fluorescence | 1.3 | 500 | 100 | 0 |
| SW620 –  Bright field | 0.9 | 0 | 1000 | - |
| SW620 –  Red Fluorescence | 1 | 500 | 100 | 0 |
